# Exercise, memory, and the hippocampus: Uncovering modifiable lifestyle reserve factors in refractory epilepsy

**DOI:** 10.1016/j.ebr.2024.100721

**Published:** 2024-10-28

**Authors:** Alena Stasenko, Erik Kaestner, Adam Schadler, Evan Brady, Jonathan Rodriguez, Rebecca W. Roth, Ezequiel Gleichgerrcht, Jonathan L. Helm, Daniel L. Drane, Carrie R. McDonald

**Affiliations:** a Department of Psychiatry, University of California, San Diego, USA; b Department of Radiation Medicine & Applied Sciences, University of California, San Diego, USA; c Department of Neurology, Emory University, USA; d Department of Neurosciences, University of California, San Diego, USA; e Department of Psychology, San Diego State University, USA; f Center for Multimodal Imaging and Genetics, University of California, San Diego, USA

**Keywords:** Epilepsy, Exercise, Physical activity, Memory, Cognition, Cognitive reserve, Resilience, Hippocampus

## Abstract

Physical exercise is an emerging target for improving cognition in aging and neurological disease. Due to the beneficial impact of exercise on hippocampal health and the vulnerability of the hippocampus in medication-resistant temporal lobe epilepsy (TLE), exercise could present a promising intervention in TLE. We investigated whether exercise engagement is associated with verbal memory function and hippocampal integrity in 29 young to middle-aged adults with refractory TLE and 21 demographically matched controls. Participants completed a self-reported questionnaire of weekly exercise, three tests of verbal memory, and a subset (*n* = 44) underwent structural MRI. Individuals with TLE self-reported lower exercise scores than controls across all levels of exercise intensity (*p* < 0.001). In TLE, greater exercise engagement was associated with better verbal memory (word-list recall and associative learning; *rho* = 0.46–0.47; *p*s _FDR_ < 0.05), and with larger contralateral hippocampal volumes (*rho* = 0.61; *p* < 0.01). These effects remained significant when controlling for epilepsy-related and demographic factors. Within the limitations of a cross-sectional observational study, these findings suggest that exercise may be a cognitive reserve factor in TLE, potentially mitigating memory decline by enhancing contralateral hippocampal integrity. With future replication and longitudinal studies to clarify the causal pathways of these relationships, exercise holds promise as a low-cost, accessible, and modifiable lifestyle target for improving cognitive health in individuals with refractory TLE.

## Highlights

• Individuals with refractory temporal lobe epilepsy (TLE) exercise less than controls.
• Greater exercise correlates with better verbal memory in TLE.
• Greater exercise correlates with contralateral hippocampal volume in TLE.
• Exercise may promote hippocampal health and improved memory in TLE.

## 1 Introduction

Memory impairment is observed in up to 50 % of adults with temporal lobe epilepsy (TLE) [1], the most common focal epilepsy, which often affects the hippocampus—a region critical to memory function. Memory problems are linked to lower functional status and a reduced quality of life [2]. However, interventions specifically targeting memory in epilepsy are lacking, with traditional cognitive rehabilitation showing limited efficacy [3]. Interestingly, common epilepsy variables such as seizure severity and duration of illness only modestly predict memory impairment [4], [5], suggesting there are undiscovered and potentially modifiable factors that could be targeted for early intervention.

Physical activity is one modifiable lifestyle factor that has been linked to various health benefits, including cognitive and brain reserve [6]. Though the mechanisms are still under investigation, exercise may increase reserve by reducing inflammation, enhancing synaptic density, improving connectivity, and boosting brain-derived neurotrophic factor (BDNF) [7]. Animal studies have shown that aerobic exercise increases hippocampal neurogenesis and long-term potentiation and learning [8], with similar benefits observed in epilepsy models [9]. Given the vulnerability of the hippocampus in TLE, exercise’s beneficial relationship with the hippocampus may make it an important intervention in this population.

However, the impact of exercise on cognitive and brain health in epilepsy is underexplored [10]. A recent systematic review [11] found preliminary evidence of the benefits of exercise on cognitive function in epilepsy across six primarily intervention studies [12], [13], [14], [10], with one being a randomized control trial [12]. However, half were in children, most focused on idiopathic generalized epilepsy (IGE), and only one directly probed memory [15]. In addition, these included heterogeneous samples, with none examining adults with medication-resistant TLE and only a couple exploring underlying mechanisms such as functional connectivity [15], [16].

Across studies, adults with epilepsy lead more sedentary lives [17], influenced by factors such as limitations from seizures and their treatment, comorbidities (e.g., mood and sleep disorders), and outdated myths and controversies about exercise increasing the likelihood of seizures [18]. Consistent with the general idea that those most deprived of exercise may derive the greatest benefit [19], it is possible that the effects of exercise on cognitive and brain health in this population may be particularly strong.

In this study, we examined the relationship between exercise, memory, and hippocampal volume in young to middle-aged adults with refractory TLE. We hypothesized that individuals with TLE would demonstrate lower levels of exercise compared to demographically matched controls, particularly for strenuous activity. Second, we predicted that for individuals with TLE, greater exercise engagement would be associated with better memory function and larger hippocampal volumes, even after accounting for established clinical and demographic factors. In contrast, these relationships may be weaker or absent in controls, as they likely have a reduced potential for exercise-related gains given a normative hippocampal volume and memory performance.

## 2 Methods

### 2.1 Participants

Twenty-nine individuals with medication-resistant TLE (*n* = 28 unilateral) undergoing presurgical evaluation and 21 controls matched for age, sex, and education were prospectively recruited as part of a larger study on imaging predictors of pre- and post-operative memory outcomes at the University of California, San Diego and Emory University (PI: McDonald). A TLE diagnosis was established by a board-certified neurologist with expertise in epileptology, in accordance with criteria defined by the International League Against Epilepsy (ILAE) with a verified temporal focus based on video-EEG. Other inclusion criteria included (1) age 18–65; (2) English-speaking; (3) absence of large space-occupying lesions (e.g., stroke). For controls, exclusion criteria included a diagnosis of a neurologic or psychiatric condition. A subset of participants (*n* = 23 TLE; *n* = 20 controls) completed structural MRI, as six individuals with TLE and one control did not undergo MRI due to time constraints or claustrophobia. The study was approved by the IRB at each site and all participants provided informed consent.

### 2.2 Physical activity measure

Self-reported weekly exercise was collected within the year of the MRI and assessed with the Godin-Shephard Leisure-Time Exercise Questionnaire; GLTEQ [20], a brief standardized tool that incorporates both intensity and frequency of current exercise. The GLTEQ was validated in healthy [21] and clinical populations, including chronic conditions [22], [23], [24], [25]. Participants were asked to report the frequency of strenuous (i.e., heart beats rapidly such as running), moderate (e.g., fast walking), or light (e.g., easy leisurely walking) activity performed for bouts of 15 min or more over a typical week. Per published work, a total Exercise Score (also termed “Leisure Index Score”) based on metabolic equivalents (METs) was calculated: (frequency [i.e., number of 15-min bouts] of strenuous activity X 9 METS + (frequency of moderate activity X 5 METs) + (frequency of light activity X 3 METs). This Exercise Score was used in all correlations. We also classified participants based on published North American guidelines for physical activity: 24 units or more = active; 14–23 = moderately active; <14 = insufficiently active/sedentary.

### 2.3 Neuropsychological measures of memory

We examined three standardized measures of verbal learning and memory: 1) word-list learning; 2) prose (i.e., stories), and 3) associative (i.e., word-pair) immediate and delayed recall, examined with the California Verbal Learning Test-2nd edition (CVLT-2), the Logical Memory (LM) subtest of the Wechsler Memory Scale-4th edition (WMS-4), and the Verbal Paired Associates (VPA) subtest of WMS-4, respectively. We used standardized scores corrected for age based on each test’s normative data.

### 2.4 Structural MRI

A subset of participants completed an MRI scan performed on a Siemens MAGNETOM Prisma 3 T scanner with a 32-channel phased-array head coil at UCSD (*n* = 19) or Emory (*n* = 25). Image acquisitions were identical at both centers and included a conventional three-plane localizer and a T1- weighted 3D structural scan (TR = 2400 msec, TE = 2.22 msec, TI = 1000 msec, flip angle 8°, FOV = 256 mm, matrix = 300 x 320, slice thickness = 0.8 mm isotropic). Automatic segmentation of the right and left hippocampus was performed with FreeSurfer (v7.3.2). Segmentations were visually inspected to ensure correct labeling of the hippocampus. To control for differences in brain size, hippocampal volume was divided by total intracranial volume. For controls, we averaged left and right hemisphere hippocampal volumes as they did not significantly differ.

### 2.5 Statistical analysis

T-tests and Chi-Square or Fisher’s Exact tests compared groups on demographic variables. A mixed ANOVA compared groups on exercise intensity levels (i.e., mild/moderate/strenuous). A multivariate ANCOVA compared groups on memory scores, controlling for education as it was significantly correlated with all memory scores. Given a smaller sample and non-normally distributed exercise scores, Spearman rank correlations were used to examine associations between exercise scores and memory and hippocampal volumes. Separate non-parametric partial correlations examined associations controlling for 1) clinical variables (i.e., mesial temporal sclerosis (MTS) status, hemisphere of seizure onset, and seizure burden [i.e., average monthly seizures X duration of illness]) and 2) demographic factors (i.e., age, education).

## 3 Results

### 3.1 Group differences in exercise (Fig. 1A)

Groups did not significantly differ in demographic variables, although controls had numerically higher education (Table 1). Exercise scores did not differ across the two sites in controls or patients (*p*s > 0.05). A 2 (Group) by 3 (Exercise Level) model revealed a significant main effect of Group of a large effect size, such that TLE had overall lower weekly exercise scores than controls [*F*(1,48) = 12.5; *p* < 0.001; *η*_p_^2^ = 0.21]. Although the interaction was not significant, the effect size was large for strenuous activity (*η*_p_^2^ = 0.25; *p* < 0.001), and medium for moderate (*η*_p_^2^ = 0.07; *p* = 0.06) and light activity (*η*_p_^2^ = 0.08; *p* = 0.04). Based on categorical cut-offs, there was a significant difference in exercise levels between groups (Fisher’s Exact = 11.1; *p* < 0.01), such that controls were more likely to be in the ‘Active’ category (86 % vs 38 %) whereas TLE were more likely to be in the ‘Insufficiently Active/Sedentary’ category (38 % vs 10 %). In TLE, exercise scores were not correlated with seizure frequency, seizure burden, number of anti-seizure medications, or age of seizure onset (*p*s > 0.05).

**Table 1:** Sample characteristics.

	TLE	Controls	*T or χ*2	*p-*value
*n*	29	21		
Age	36.4 (10.3)	34.8 (16.6)	−0.40	0.70
Education	14.3 (2.5)	15.6 (2.5)	1.8	0.07
Sex (female)	14 (67 %)	21 (72 %)	0.19	0.76
Handedness (right)	27 (93 %)	19 (91 %)	1.5	0.75
Ethnicity (non-Hispanic)	23 (79 %)	19 (91 %)	1.1	0.44
Race			5.0	0.32
White	19 (66 %)	10 (47.6 %)		
Black or African American	6 (21 %)	4 (19 %)		
Asian	1 (3 %)	4 (19 %)		
More than one race or Other	3 (10 %)	2 (9.5 %)		
Am. Indian/Alaska Native	0 (0 %)	1 (4.8 %)		
Age of seizure onset	23.2 (10.5)	—		
Side of seizure onset (L/R/B)	16/12/1	—		
Epilepsy duration (years)	13.2 (12.3)	—		
Seizure frequency (per month)	9.3 (11.3)	—		
Seizure burden*	81.2 (92.0)	—		
Number of ASMs	2.8 (1.2)	—		
MTS status (No)	18 (62 %)	—		

*Note*: L = left; R = right; B = bilateral; ASM = anti-seizure medication; MTS = mesial temporal sclerosis; *Seizure burden = # of seizures reported per month X duration of illness (years).

**Fig. 1:**
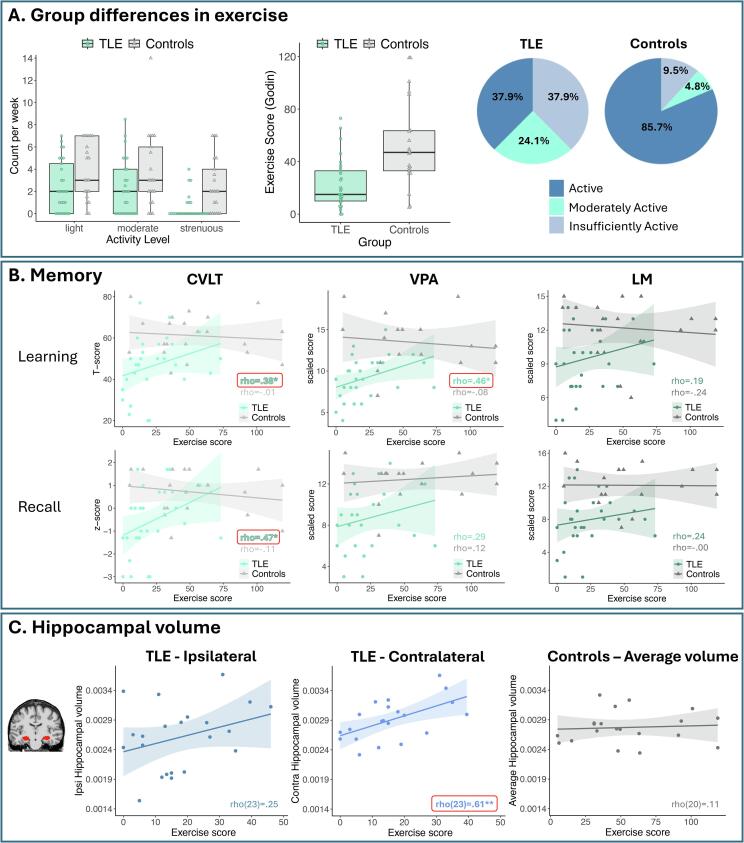
**(A)** Group differences in self-reported exercise levels. First panel plots weekly light, moderate, and strenuous activity by group. Middle panel plots total weekly Exercise Scores based on the GLTEQ. Right panel shows percentages of exercise levels derived from standardized cut-offs from the GLTEQ. (**B)** Spearman bivariate correlations between total Exercise Score and learning and recall performance on CVLT (left panel), VPA (middle panel), and LM (right panel), with significant correlations bolded and in red boxes. (**C)** Spearman bivariate correlations between total Exercise Scores and ipsilateral and contralateral hippocampal volume in TLE (left and middle panels) and average hippocampal volume in controls (right panel), with significant correlations bolded and in red boxes.

### 3.2 Group differences in memory and hippocampal volume

An effect of site was not present in memory scores nor hippocampal volumes for controls or TLE (*p*s > 0.05). TLE overall scored lower than controls on all six memory measures, controlling for education (overall MANCOVA: *F* = 4.8; *p* = 0.001; *η*_p_^2^ = 0.47; individual *p*s < 0.05). Ipsilateral and contralateral hippocampal volumes did not differ in TLE versus controls (*p*s > 0.05).

### 3.3 Associations between exercise and memory function (Fig. 1B)

#### 3.3.1 Controls

In controls, correlations between exercise scores and memory scores were not statistically significant in any measure (*p*s > 0.05)

#### 3.3.2 TLE

In TLE, higher exercise scores were associated with higher performance on CVLT Learning [*rho*(28) = 0.38; *p* = 0.049], CVLT delayed recall [*rho*(28) = 0.47; *p* = 0.01]; and VPA immediate recall [*rho*(28) = 0.46; *p* = 0.01], of which all survived a 5 % FDR correction for multiple comparisons except for CVLT Learning, which became marginally significant after correction (*p* = 0.09). The correlations for LM did not reach significance (*p*s > 0.05).

#### 3.3.3 Control for clinical and demographic variables

We examined whether the above associations remained significant after controlling for clinical variables known to contribute to verbal memory impairment in TLE—hemisphere of seizure onset, MTS status, and seizure burden (see 2.5). With these three covariates entered simultaneously, correlations remained significant and of a similar magnitude for CVLT delayed recall [*r*(22) = 0.46; *p* = 0.03] and VPA immediate recall [*r*(22) = 0.47; *p* = 0.02), whereas the association with CVLT learning became marginally significant [*r*(22) = 0.37; *p* = 0.07]. In a separate partial correlation controlling for age and education, CVLT delayed recall [*r*(25) = 0.40; *p* = 0.04] and VPA immediate recall [*r*(25) = 0.42; *p* = 0.03] remained significantly associated with exercise scores, whereas CVLT learning was no longer significant [*r*(25) = 0.27; *p* = 0.18].

### 3.4 Associations between exercise and hippocampal volume (Fig. 1C)

#### 3.4.1 Controls

In controls, correlations between exercise scores and hippocampal volume were not significant for average, left, or right hemisphere volumes (*p*s > 0.05).

#### 3.4.2 TLE

In TLE, higher exercise scores were associated with greater contralateral hippocampal volume [*rho*(23) = 0.61; *p* = 0.002]. For ipsilateral hippocampal volume, this association was positive but not statistically significant [*rho(*23) = 0.25; *p* = 0.26]**.**

#### 3.4.3 Control for clinical and demographic variables

The correlation between contralateral hippocampal volume and exercise score remained statistically robust when simultaneously controlling for hemisphere of seizure onset, MTS status, and seizure burden [*r*(18) = 0.65; *p* = 0.002]. In addition, this correlation remained significant when controlling for age and education [*r*(18) = 0.61; *p* = 0.004].

## 4 Discussion

Exercise has emerged as a promising modifiable lifestyle factor that may be used in the clinical toolkit to preserve cognition in aging and neurological diseases. For example, physical activity is now incorporated into dementia prevention guidelines [19]. Preliminary data in epilepsy suggests exercise may also benefit cognitive function in this population [11], but important gaps must be filled such as investigating medication-resistant (i.e., refractory) TLE, biological mechanisms, and additional cognitive domains. Here, we examined whether self-reported exercise in younger-to-middle-aged adults with refractory TLE is associated with verbal memory function and hippocampal structure, both of which are vulnerable in mesial TLE. Individuals with TLE reported lower levels of exercise than demographically matched controls across all intensity levels. Second, greater exercise engagement was associated with better verbal memory performance (i.e., word-list recall and associative word-pair learning) in TLE only. Third, higher exercise was associated with larger contralateral hippocampal volumes in TLE. Together, these findings suggest that exercise may be associated with improved hippocampal health and verbal memory function in TLE. As these correlations are based on cross-sectional data, this should be examined in future longitudinal and interventional studies, and with causal (e.g., mediation) approaches.

### 4.1 Reduced exercise levels in refractory TLE

Our finding of lower exercise levels in TLE compared to controls is concerning, and consistent with early studies showing increased sedentary behavior in other epilepsy populations [26]. We extend these previous reports to a homogenous group of young to middle-aged adults with medication-resistant TLE. Notably, we observed a trend toward more pronounced reductions in higher exercise intensities (e.g., running), though the interaction was not significant. Reduced exercise in this population may be due to a host of factors such as fear of inducing seizures during exercise, side effects from anti-seizure medications, transportation barriers, and multiple co-morbidities (e.g., mood and sleep disorders). Our sample of individuals with refractory TLE may face additional challenges to engagement in exercise, including uncontrolled seizures and the burden of frequent medical appointments and procedures, perhaps leading to a more sedentary lifestyle. Though we did not inquire about barriers to exercise in this study, this would be a fruitful future investigation. Despite a group-level reduction in exercise, we observed high variability in exercise levels, with a significant portion of our sample (62 %) at least moderately active, indicating that many individuals with TLE can and do continue to engage in physical activity. This is promising, as the latest ILAE report on this issue indicates that beyond other benefits, physical exercise may favorably affect seizure control and provides specific guidelines for safe exercise options for those with uncontrolled seizures [18].

### 4.2 Exercise and verbal memory in TLE

The observed associations between exercise and performance on several memory measures is suggestive of cognitive benefits of exercise in epilepsy [11], consistent with studies in adults [12], [27] and children [14] in IGE that found benefits primarily in executive function and attention. Our findings closely align with a previous study that examined memory in IGE [15]. This pilot randomized control trial found improvements in CVLT performance in the exercise but not control group, which was associated with increased functional connectivity [15]. Importantly, the associations that we observed in TLE remained significant after controlling for traditional reserve variables such as education, as well as clinical variables such as seizure burden, hemisphere of seizures, and MTS status. This suggests that exercise may independently contribute to memory function in TLE, over and above known factors. However, we were unable to examine the relative contributions of these variables in one large model due to limited power and a smaller sample. In addition, our finding of an absence of associations in controls suggests that the benefits of exercise on memory may be more pronounced in TLE, potentially due to the unique impact of seizures on memory systems. That is, we speculate that the benefits of exercise are only apparent in the group with a biological vulnerability, for which exercise is protective. However, it is also possible that a restricted range of memory scores and hippocampal volumes could have contributed to null effects in controls. Indeed, previous evidence has suggested positive effects of exercise on memory function even in healthy young to middle-aged adults [28].

Interestingly, while we observed associations for word-list and associative learning, we did not find significant effects for prose memory. Though we interpret a null effect cautiously, prose memory is thought to be less dependent on hippocampal and medial temporal structures [29]. Therefore, this pattern of findings is expected under the assumption that the hippocampus may show the greatest benefits of exercise and is most disrupted in mesial TLE.

### 4.3 Exercise and hippocampal reserve

Our findings suggest a potential role of exercise in enhancing structural reserve, particularly in the healthier hippocampus. That is, consistent with the role of the hippocampus in memory and exercise, we found novel evidence linking exercise engagement to the integrity of the *contralateral* hippocampus—typically less prominently affected in the epileptic network in unilateral TLE. This was true even when controlling for important variables that could also affect brain reserve such as age, education and seizure burden. The ipsilateral hippocampus showed a similarly positive, albeit not statistically robust trend. Though primarily examined in animal models [7], exercise is known to increase hippocampal neurogenesis, particularly in the dentate gyrus [8], potentially leading to increased gray matter volume. Other proposed mechanisms include improved cerebral blood flow, reduced oxidative stress, enhanced functional connectivity, and increased synaptic plasticity, mediated by higher levels of BDNF, a protein that supports neuronal growth and function [7]. Though these mechanisms require in-depth investigation in epilepsy, our preliminary findings may reflect a reserve mechanism in TLE, with exercise potentially contributing to the structural integrity of the less affected hippocampus by stimulating beneficial neuroplasticity.

### 4.4 Limitations and future directions

Our observational design limits the ability to infer causality, and the directionally of effects remain unclear (i.e., an alternative explanation being that lower memory function leads to less exercise engagement). In addition, although we controlled for several factors related to epilepsy severity given that greater severity may lead to less exercise, longitudinal designs are needed to tease apart the role of seizure burden in exercise participation. Our sample size was modest, though well-characterized and geographically and racially diverse. Nonetheless, findings require replication in larger samples and with multivariate models to better understand the unique contributions of exercise, in relation to other known factors of reserve. As our sample was primarily female, future investigation of possible sex differences [30] in these relationships is warranted. We focused on verbal memory and hippocampal volume due to their relevance in medial TLE, but future studies should examine the effects of exercise on other cognitive domains and extra-hippocampal networks. In addition, other factors such as cerebrovascular risk, white matter integrity, psychiatric comorbidities, and substance use may interact with exercise engagement as well as memory function and hippocampal integrity. Although we were unable to examine their influence on outcomes in the current study, this should be considered in future research.

Self-reported exercise levels are known to be less accurate than objective measures, which was previously confirmed in an epilepsy sample [31]. Future research should incorporate objective measures such as accelerometers or Fitbit-type devices to improve data reliability, allow for continuous tracking, and provide a more nuanced understanding of the relationship between exercise and cognitive and brain health in epilepsy. For example, it remains unknown what levels of exercise intensity are needed to achieve benefits. We were unable to test this given a restricted range of strenuous exercise reported in TLE. Finally, although the GLTEQ is well-validated and measures both frequency and intensity of activity, it is not as commonly used, as for example, the International Physical Activity Questionnaire (IPAQ) [32] which additionally incorporates duration of activity (e.g., in hours or minutes). Finally, the GLTEQ questionnaire captures only recent exercise activity. Long-term exercise over months or years could potentially have a greater cumulative impact on hippocampal volume and memory function due to sustained engagement. Future studies should consider incorporating measures of premorbid and longer-term duration of exercise, particularly for chronic conditions like TLE.

## 5 Conclusion

To date, the primary clinical target to improve cognitive comorbidities in epilepsy has been seizure control. Our findings, though cross-sectional, combined with interventional studies suggest that a modifiable lifestyle factor like exercise may benefit verbal memory function and hippocampal integrity in young to middle-aged adults with refractory TLE, acting as a cognitive reserve factor. As exercise can be low-cost, accessible, and tailored to individuals, it holds promise as a targeted intervention to mitigate memory decline in this population. Beyond cognition, physical activity positively impacts mood and quality of life in epilepsy [31], [33] and warrants further investigation into its broader benefits.

## Ethical Statement

The authors confirm the following:

• The work described has not been published previously except in the form of a preprint, an abstract, a published lecture, academic thesis or registered report.
• The article is not under consideration for publication elsewhere.
• The article’s publication is approved by all authors and tacitly or explicitly by the responsible authorities where the work was carried out.
• if accepted, the article will not be published elsewhere in the same form, in English or in any other language, including electronically without the written consent of the copyright-holder.

## CRediT authorship contribution statement

**Alena Stasenko:** Writing – original draft, Formal analysis, Conceptualization. **Erik Kaestner:** Writing – review & editing, Conceptualization. **Adam Schadler:** Data curation. **Evan Brady:** Project administration, Data curation. **Jonathan Rodriguez:** Project administration, Conceptualization. **Rebecca W. Roth:** Methodology, Data curation. **Ezequiel Gleichgerrcht:** Writing – review & editing, Methodology. **Jonathan L. Helm:** Formal analysis. **Daniel L. Drane:** Resources, Data curation. **Carrie R. McDonald:** Writing – review & editing, Funding acquisition, Conceptualization.

## Declaration of competing interest

The authors declare the following financial interests/personal relationships which may be considered as potential competing interests: Dr. McDonald is a consultant for Neurona Therapeutics. The remaining authors do not have any conflicts of interest to disclose.
